# Long-Term, Patient-Level Analysis of Radiofrequency Renal Denervation in the SYMPLICITY Clinical Trial Program

**DOI:** 10.1016/j.jacadv.2025.101606

**Published:** 2025-02-21

**Authors:** Felix Mahfoud, Raymond R. Townsend, David E. Kandzari, Giuseppe Mancia, Robert Whitbourn, Lucas Lauder, Deepak L. Bhatt, Kazuomi Kario, Roland E. Schmieder, Markus Schlaich, Martin Fahy, Michael Böhm

**Affiliations:** a Department of Cardiology, University Heart Center, University Hospital Basel, Basel, Switzerland; b Cardiovascular Research Institute Basel (CRIB), University Heart Center, University Hospital Basel, Basel, Switzerland; c Perelman School of Medicine, University of Pennsylvania, Philadelphia, Pennsylvania, USA; d Piedmont Heart Institute, Atlanta, Georgia, USA; e Department of Clinical Medicine and Prevention, University of Milano-Bicocca, Milan, Italy; f Department of Cardiology, St Vincent's Heart Centre, Melbourne, Australia; g Mount Sinai Fuster Heart Hospital, Icahn School of Medicine at Mount Sinai, New York, New York, USA; h Department of Cardiovascular Medicine, Jichi Medical University School of Medicine, Shimotsuke, Tochigi, Japan; i Department of Nephrology and Hypertension, University Hospital Erlangen, Erlangen, Germany; j Dobney Hypertension Centre, Medical School, Royal Perth Hospital Unit, Perth, Australia; k Medtronic, Santa Rosa, California, USA; l Universitatsklinikum des Saarlandes, Klinik für Kardiologie, Angiologie und Internistische Intensivmedizin, Universitätsklinikum des Saarlandes, Saarland University, Homburg, Germany

**Keywords:** hypertension, linear regression model, renal denervation

## Abstract

**Background:**

Renal denervation (RDN) lowers blood pressure (BP) in patients with uncontrolled hypertension. Current guidelines recommend RDN for patients with uncontrolled BP despite the use of antihypertensive (AH) medications. Durability of BP reductions and assessment of which patient baseline characteristics correlate with subsequent BP reductions are scarce.

**Objectives:**

The authors leveraged patient data from the entire SYMPLICITY Clinical program to model long-term BP reductions and assess patient characteristics associated with future BP reductions.

**Methods:**

Repeated BP measurements from each patient were analyzed using linear mixed models. Models were fitted with office systolic BP (SBP), 24-h ambulatory SBP, office diastolic BP (DBP), and 24-h ambulatory DBP as outcome variables. Baseline BP, baseline number of AH medications, AH medications over time, and other variables were included as fixed effects.

**Results:**

The mixed model included data from 4,155 patients treated with the Symplicity RDN system. The mean age was 60 ± 12 years, 40.4% of whom were female. Estimated, longitudinal office and 24-h ambulatory SBP changes through 36 months, after adjusting for AH medication effects, were biphasic, with a steep reduction after RDN through the first 6 months followed by continuous and steady reductions in office and 24-h SBP and DBP afterward through 36 months. Higher baseline office systolic or 24-h ambulatory SBP were correlated with greater reductions through follow-up in office and 24-h SBP, respectively. Patient characteristics consistent with high sympathetic nerve activity, such as atrial fibrillation and type 2 diabetes, emerged as statistically significant covariates associated with greater office systolic and office and 24-h diastolic BP reductions, respectively.

**Conclusions:**

Modeling suggested patients have durable BP reductions following RDN, with a steep immediate reduction followed by a steady reduction through 3 years.

Hypertension is the leading modifiable cause of death worldwide, affecting more than 15% of the global population.^1^^,^^2^ Reducing blood pressure (BP) lowers the risk of cardiovascular events and mortality.^3^^,^^4^ Catheter-based renal denervation (RDN) has emerged as a new treatment option for uncontrolled hypertension according to current guidelines and is the first U.S. Food and Drug Administration-approved device-based therapy for uncontrolled hypertension.^5^^,^^6^ Multiple clinical randomized control trials and all-comer registries have demonstrated the safety and efficacy of the procedure.^7^, ^8^, ^9^, ^10^, ^11^ However, individual studies have unique inclusion criteria, study populations, and trial designs with focused primary endpoint collection at 6 months or less after the procedure. Comprehensive analyses are warranted to gain better insight into the applicability of RDN as a suitable therapy for a wide range of patients with uncontrolled hypertension representative of real-world scenarios. Moreover, data regarding durability of the procedure are important to assess the value of RDN in clinical practice.

The international SYMPLICITY Clinical program is the largest of its kind with a wide spectrum of 4,155 patients treated with RDN for uncontrolled hypertension. We leveraged this large, pooled patient-level data set to develop a multivariate, mixed model to estimate long-term BP changes after radiofrequency (RF) RDN through 3 years (Central Illustration). Furthermore, we assessed whether specific patient characteristics, singly or in combination, are associated with future BP reductions.

**Central Illustration:**
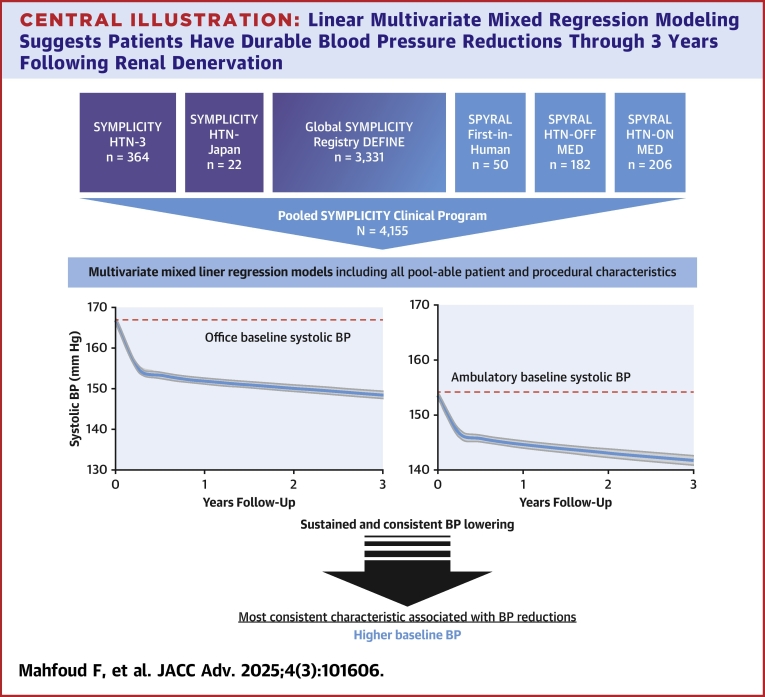
Linear Multivariate Mixed Regression Modeling Suggests Patients Have Durable Blood Pressure Reductions Through 3 Years Following Renal Denervation RDN patient data (n = 4,155) from the entire SYMPLICITY clinical program was leveraged to model long-term BP reductions after the procedure and assess patient characteristics associated with future BP reductions. Higher baseline BP was the most consistent patient characteristic significantly associated with clinically relevant BP reductions after RDN. Further, prospective studies are needed to validate whether other patient characteristics are associated with response. Abbreviations as in Figure 1.

## Methods

### Study designs and patient population

The program includes patients treated with RF RDN from multiple randomized, sham-controlled trials such as SYMPLICITY HTN-3 (Renal Denervation in Patients With Uncontrolled Hypertension), SPYRAL HTN-OFF MED (Global Clinical Study of Renal Denervation With the Symplicity Spyral Multi-electrode Renal Denervation System in Patients With Uncontrolled Hypertension in the Absence of Antihypertensive Medications), and SPYRAL HTN-ON MED (Global Clinical Study of Renal Denervation With the Symplicity Spyral Multi-electrode Renal Denervation System in Patients With Uncontrolled Hypertension on Standard Medical Therapy); the randomized (but not sham-controlled) study, HTN-Japan; SPYRAL First-In-Human, a feasibility study; and the Global SYMPLICITY Registry (GSR) DEFINE, a global all-comers study reflecting a real-world population. Details of the included component study designs have been published previously.^12^, ^13^, ^14^, ^15^, ^16^, ^17^ Briefly, SYMPLICITY HTN-3 and HTN-Japan were prospective, blinded, multicenter, randomized trials to assess the safety and efficacy of RDN using the first-generation Symplicity Flex single-electrode, RF catheter. Patients were required to have an office systolic BP ≥160 mm Hg and 24-h ambulatory SBP ≥135 mm Hg despite at least 3 antihypertensive (AH) medications. SPYRAL First-In-Human was a feasibility study of the Symplicity Spyral multielectrode RF RDN catheter. To be eligible, patients were required to have an office SBP ≥160 mm Hg or ≥150 mm Hg for those with type 2 diabetes, despite prescription of ≥3 AH medication classes. SPYRAL HTN-OFF MED and SPYRAL HTN-ON MED were global, randomized, blinded, sham-controlled trials designed to assess the safety and efficacy of RF RDN in the absence or presence of AH medications, respectively. To be eligible, patients with uncontrolled hypertension and between 18 and 80 were required to have an office SBP ≥150 and <180 mm Hg, an office diastolic BP ≥90 mm Hg, and a 24-h ambulatory SBP ≥140 and <170 mm Hg. The OFF MED trial required a washout period from AH medications and enrolled patients remained off medications through the first 3 months. ON MED trial patients were prescribed a stable regimen of 1 to 3 AH medications 6 weeks prior to randomization, and through 6 months of follow-up. GSR DEFINE is a prospective, multicenter, single-arm, open-label, observational, international registry to evaluate the safety and efficacy of RF RDN in an all-comer, real-world population. Sham control patients from the randomized control trials who later crossed over to receive the RDN procedure were not included in this analysis. Each study was approved by the local Institutional Review Boards or Ethics Committees, and all patients provided written informed consent.

### Procedure

Procedural details have been published previously.^7^^,^^8^^,^^13^^,^^17^ Briefly, the procedure was performed utilizing either the Symplicity Flex single-electrode or the Symplicity Spyral multielectrode RDN catheter (Medtronic) and Symplicity G3 RF generator (Medtronic) to provide circumferential RF ablations of the renal arteries and all accessible branch vessels 3 to 8 mm in diameter. Cases were all performed by an experienced proceduralist and, in the case of randomized controlled trials, were proctored according to predetermined treatment plans.

### Follow-up

Patients were followed at 3, 6, 12, 24, and 36 months postprocedure for all studies. Office and 24-h ambulatory BP were obtained at each follow-up. Incidence of renal artery stenosis, stroke, death, cardiovascular death (including unknown death), myocardial infarction, and hospitalization for a hypertensive crisis were recorded at each follow-up.

### Statistical analyses

Continuous variables are expressed as mean ± SD. Categorical variables are expressed as percent. Continuous repeated measurements were analyzed using linear mixed effects models incorporating every available BP measurement from every patient from baseline through 36 months. Mixed models, incorporating both fixed and random effects, were fitted with office and ambulatory systolic and diastolic BP as outcome variables. Fixed effects in the models were baseline BP (intervals of 10 mm Hg), prescribed AH medication classes, number of medications at baseline, and over time, and baseline characteristics including age, sex, history of myocardial infarction (MI), type 2 diabetes, body mass index (BMI), sleep apnea, smoking history, heart failure, atrial fibrillation, baseline estimated glomerular filtration rate (eGFR), baseline serum creatinine, procedure duration (10 min intervals), catheter time (10 min intervals), contrast volume (10 mL intervals), and number of ablations (intervals of 4 since each placement of the Spyral catheter equals 4 or fewer ablations). Certain patient characteristics that were highly correlative were not included to avoid artefacts in the model, such as baseline pulse pressure and heart rate, which were found to be highly correlated with baseline BP. Models of ambulatory BP include baseline office BP as well as a covariate. Study and subject were treated as nested random effects and modeled using an unstructured covariance matrix. Models were rerun including only significant fixed effects to identify patient characteristics that were associated with significant changes in BP at follow-up. Least square means approach was used to estimate the follow-up BP measurements at each follow-up visit from the models. Multicollinearity was assessed using variance inflation factors, calculated for all candidate fixed effect variables for models of office and 24-h SBP and DBP change through 36 months. Statistical analysis was performed using SAS for Windows 9.4 (SAS Institute).

## Results

### Baseline demographics

The mixed, multivariate analysis includes data from 4,155 patients from the SYMPLICITY RDN program who underwent RF RDN. RDN was performed using either the Symplicity Flex (n = 2,616, 63%) or Spyral catheter (n = 1,539, 37%). Baseline patient characteristics are provided in Table 1. The mean age was 60 ± 12 years, 40.4% of whom were female. The mean baseline office SBP was 166.4 ± 23.6 mm Hg with a corresponding mean baseline 24-h ambulatory SBP of 154.1 ± 17.3 mm Hg, respectively. The average number of prescribed AH medications at baseline was 4.5 ± 2.0. The AH medication classes prescribed at baseline are provided in Supplemental Table 1. Among pooled patients, 8.4% had a history of MI, 35.8% had type 2 diabetes, 20.6% had sleep apnea, 10.8% had atrial fibrillation, and 35.0% had a history of smoking. At baseline, mean eGFR was 76.5 ± 24.0 ml/min/1.73 m^2^, with 22.9% having an eGFR below 60 ml/min/1.73 m^2^, mean BMI was 31.3 ± 6.2 kg/m^2^, mean office heart rate was 70.7 ± 13.0 bpm, and mean 24-h ambulatory heart rate was 69.8 ± 11.7 bpm. Procedural characteristics are detailed in Supplemental Table 2.

**Table 1:** Baseline Patient Characteristics From the SYMPLICITY RDN Program

	All Patients (N = 4,155)	Spyral Patients (n = 1,539)	Flex Patients (n = 2,616)
Male	59.6% (2,476/4,155)	61.8% (951/1,539)	58.3% (1,525/2,616)
Age (y)	59.5 ± 12.0 (4,155)	58.1 ± 12.3 (1,539)	60.4 ± 11.7 (2,616)
BMI (kg/m^2^)	31.3 ± 6.2 (4,111)	31.0 ± 6.7 (1,519)	31.4 ± 5.9 (2,592)
eGFR (mL/min/1.73 m^2^)	76.5 ± 24.0 (3,963)	77.9 ± 24.4 (1,493)	75.7 ± 23.7 (2,470)
eGFR <60 mL/min/1.73 m^2^	22.9% (907/3,963)	21.4% (322/1,503)	23.7% (585/2,470)
eGFR <45 mL/min/1.73 m^2^	8.6% (340/3,963)	8.8% (132/1,503)	8.4% (208/2,470)
Serum creatinine (mg/dL)	1.1 ± 0.9 (3,963)	1.1 ± 1.1 (1,493)	1.1 ± 0.8 (2,470)
Aldosterone (ng/dL)	9.6 ± 6.9 (329)	8.6 ± 6.3 (172)	10.8 ± 7.4 (157)
Sodium (mmol/L)	140.0 ± 3.2 (3,078)	140.1 ± 2.8 (984)	139.9 ± 3.3 (2094)
Potassium (mmol/L)	4.1 ± 0.5 (3,101)	4.2 ± 0.5 (987)	4.1 ± 0.5 (2,114)
Previous myocardial infarction	8.4% (307/3,663)	5.6% (60/1,067)	9.5% (247/2,596)
Type 2 diabetes mellitus	35.8% (1,481/4,132)	30.1% (458/1,522)	39.2% (1,023/2,610)
Heart failure	10.2% (373/3,663)	6.0% (64/1,067)	11.9% (309/2,596)
Sleep apnea	20.5% (799/3,888)	18.5% (269/1,456)	21.8% (530/2,432)
Atrial fibrillation	10.8% (446/4,124)	8.3% (127/1,522)	12.3% (319/2,602)
Smoker	35.0% (1,452/4,145)	36.1% (553/1,531)	34.4% (899/2,614)
Office systolic BP (mm Hg)	166.4 ± 23.6	164.3 ± 22.3	167.6 ± 24.3
Office diastolic BP (mm Hg)	91.7 ± 16.3	93.4 ± 15.5	90.8 ± 16.7
Office pulse pressure (mm Hg)	74.7 ± 20.3	71.0 ± 19.8	76.9 ± 20.3
24-h ambulatory systolic BP (mm Hg)	154.1 ± 17.3	152.4 ± 16.8	155.1 ± 17.5
24-h ambulatory diastolic BP (mm Hg)	88.2 ± 16.3	90.4 ± 13.5	86.8 ± 14.1
Daytime systolic BP (mm Hg)	157.1 ± 17.4	155.7 ± 16.7	158.0 ± 17.8
Nighttime systolic BP (mm Hg)	146.3 ± 20.2	144.1 ± 19.2	147.6 ± 20.6
24-h pulse pressure (mm Hg)	65.9 ± 14.8	62.0 ± 15.0	68.2 ± 14.2
Office heart rate (beats/min)	70.7 ± 13.0	72.1 ± 12.6	69.9 ± 13.2
24-h ambulatory heart rate (beats/min)	69.8 ± 11.7	71.4 ± 11.4	68.9 ± 11.7
Baseline number of antihypertensive medications (prescribed)	4.5 ± 2.0	3.8 ± 2.3	5.0 ± 1.6
Baseline number of antihypertensive medications (prescribed)	5.0 (3.0-6.0)	4.0 (2.0-5.0)	5.0 (4.0-6.0)

Values are % (n/N), mean ± SD (N), mean ± SD, or median (IQR).

BMI = body mass index; BP = blood pressure; eGFR = estimated glomerular filtration rate; RDN = renal denervation.

### Antihypertensive medications over time

Through 36 months after RDN, the mean number of AH medications did not change (4.5 ± 2.0 compared with 4.5 ± 2.0 at baseline, *P* = 0.33). Modeled least square mean estimates for BP changes over time are adjusted for medications at each follow-up.

### Long-term blood pressure changes

**Figure 1:**
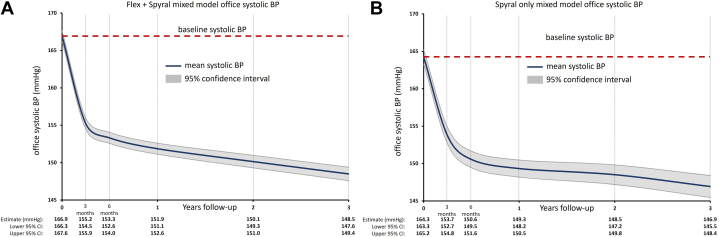
Mixed Model Office Systolic Blood Pressure Estimates After Radiofrequency Renal Denervation Using the Symplicity Radiofrequency Renal Denervation System Through 36 Months Least square means estimates of office systolic BP from baseline through 36 months after RF RDN from patients treated with either the (A) flex and spyral catheter or (B) the spyral catheter only. BP = blood pressure; RDN = renal denervation; RF = radiofrequency.

**Figure 2:**
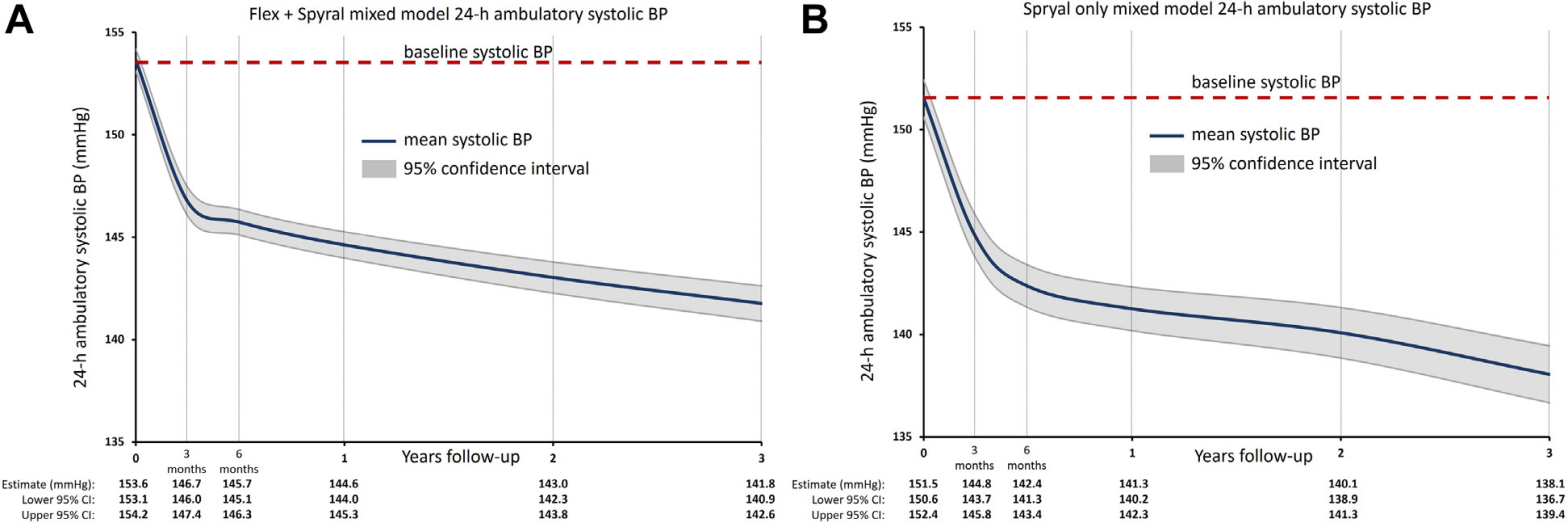
Mixed Model 24-h Ambulatory Systolic Blood Pressure Estimates After Radiofrequency Using the Symplicity Radiofrequency Renal Denervation System Through 36 Months Least square means estimates of 24-h ambulatory systolic BP from baseline through 36 months after RF RDN from patients treated with either the (A) flex and spyral catheter or (B) the spyral catheter only. Abbreviations as in Figure 1.

Continuous repeated measurements were analyzed using linear mixed effects models incorporating every available BP measurement from every patient from baseline through 36 months. The mean follow-up duration was 854 ± 408 days, with a median of 1,076 days. Least square means estimates from the model show sustained office and 24-h ambulatory SBP reductions from baseline through 36 months. Estimated office SBP reduction over time was biphasic (Figure 1), with a steeper BP reduction immediately following RDN through the first 3 to 6 months, followed by a steadier BP reduction through 36 months. The 24-h ambulatory SBP was also biphasic (Figure 2) through 36 months for both the Flex and Spyral mixed model and the Spyral-only model. Least square means estimates from the model also show sustained office and 24-h ambulatory DBP reductions, with a similar initial steeper reduction through 6 months, followed by a steadier reduction through 36 months (Supplemental Figures 1 and 2). Measured mean office and 24-h ambulatory pulse pressure among pooled patients from the Flex and Spyral cohorts declined over time (Supplemental Figure 3).

### Patient characteristics and associated long-term BP changes

Higher baseline BP was associated with the largest BP reduction through follow-up (Table 2). Combined hypertension (a patient with both systolic and diastolic hypertension) was associated with office and 24-h ambulatory SBP reductions. In contrast, an increased number of medications prescribed at baseline was associated with a higher office and 24-h ambulatory SBP at follow-up. Several parameters were associated with office SBP reductions but not 24-h SBP reductions. History of heart failure, history of atrial fibrillation, and baseline serum creatinine were each independently associated with significantly greater office SBP reductions (Table 2). In contrast, prescription of a vasodilator was associated with less favorable office SBP reductions at follow-up compared with patients not prescribed a vasodilator. Characteristics independently associated with 24-h ambulatory SBP reductions were increased age, prescription of an aldosterone antagonist, and baseline eGFR (Table 3). Reductions in office and 24-h ambulatory SBP through 36 months after RDN were independent of predefined subgroups including sex, history of myocardial infarction and diabetes, BMI, sleep apnea, smoking history, procedural duration, catheter time, number of ablations, and contrast volume (Supplemental Table 3). Patient characteristics associated with office and 24-h ambulatory DBP reductions are shown in Supplemental Tables 4 and 5.

**Table 2:** Modeling Results for Fixed Effects on Office Systolic Blood Pressure Change Through 36 Months

Fixed Effect	SBP Change Estimate (mm Hg)	95% CI (mm Hg)	Pr > |t|
Baseline office systolic BP (+10 mm Hg)	−4.52	−4.73 to −4.32	<0.0001
No. of medications at baseline	0.51	0.28-0.74	<0.0001
History of heart failure	−1.64	−3.04 to −0.24	0.021
History of atrial fibrillation	−1.54	−2.92 to −0.16	0.029
Combined hypertension	−1.16	−2.13 to −0.20	0.018
Baseline serum creatinine (+1 mg/dL)	−0.75	−1.26 to −0.24	0.0040
Prescribed vasodilator	3.09	1.81-4.36	<0.0001
Follow-up			
Baseline	Reference	-	-
3 mo	−11.76	−10.89 to −12.63	<0.0001
6 mo	−13.64	−12.77 to −14.51	<0.0001
12 mo	−15.08	−14.20 to −15.96	<0.0001
24 mo	−16.80	−15.85 to −17.75	<0.0001
36 mo	−18.45	−17.43 to −19.46	<0.0001

Nonsignificant covariates excluded.

SBP = systolic blood pressure; other abbreviation as in Table 1.

**Table 3:** Modeling Results for Fixed Effects on 24-h Ambulatory Systolic Blood Pressure Change Through 36 Months

Fixed Effect	SBP Change Estimate (mm Hg)	95% CI (mm Hg)	Pr > |t|
Baseline ambulatory systolic BP (+10 mm Hg)	−3.59	−3.83 to −3.34	<0.0001
Baseline office systolic BP (+10 mm Hg)	0.82	0.62-1.02	<0.0001
No. of medications at baseline	1.29	0.98-1.61	<0.0001
No. of medications through follow-up	−0.89	−1.17 to −0.61	<0.0001
Increased age (per year)	−0.05	−0.09 to −0.02	0.0034
Combined hypertension	−1.79	−2.72 to −0.86	0.0002
Prescribed aldosterone antagonist	−1.32	−2.31 to −0.33	0.0089
Baseline eGFR (mL/min/1.73 m^2^)	−0.02	−0.04 to −0.002	0.030
Follow-up			
Baseline	Reference	-	-
3 mo	−6.95	−7.72 to −6.17	<0.0001
6 mo	−7.91	−8.63 to −7.20	<0.0001
12 mo	−9.01	−9.76 to −8.27	<0.0001
24 mo	−10.61	−11.45 to −9.76	<0.0001
36 mo	−11.87	−12.80 to −10.95	<0.0001

Nonsignificant covariates excluded.

Abbreviations as in Tables 1 and 2.

As a sensitivity analysis, characteristics associated with BP change in a mixed model specific to the Spyral device were also assessed. As with the comprehensive model, atrial fibrillation and baseline serum creatinine were associated with larger office systolic reductions in the “Spyral-only” model (Supplemental Table 6). Combined hypertension, baseline serum potassium, and an increase in the number of ablations were associated with greater 24-h SBP reductions (Supplemental Table 7). Increased age and baseline serum creatinine were also associated with greater office DBP reductions (Supplemental Table 8). In addition, baseline eGFR and potassium were associated with greater office DBP reductions. Greater 24-h ambulatory DBP reductions were associated with higher baseline serum potassium levels (Supplemental Table 9). We tested for multicollinearity among fixed effect variables (see Methods). Overall, there was no evidence for strong multicollinearity among the fixed effect variables included in the final models.

### Clinical outcomes

Safety events were rare through 3 years after RDN (Supplemental Table 10). Only 6 patients experienced renal artery stenosis (0.3%), however there were no instances of significant renal artery stenosis or stent implantations at any time following RDN with the latest-generation Spyral RDN catheter. The rate of death, cardiac death, myocardial infarction, and stroke at 3 years was 5.1%, 2.8%, 2.7%, and 4.5%, respectively, consistent with a high risk, aging population with multiple comorbidities. The rate of hospitalization for hypertensive crisis through 3 years was 3.5%. Additional evaluation limited only to patients who underwent RDN using the next-generation Spyral catheter revealed similarly low rates (Supplemental Table 10). Event rates through 1 year are provided in Supplemental Table 11.

## Discussion

In this multivariate mixed model utilizing the largest RDN patient-level clinical program to date, office and 24-h ambulatory systolic and diastolic BP reductions were sustained through 3 years. BP reductions were biphasic with an amplified change over time. Pool-able, patient and procedural characteristics were also incorporated into the model to assess whether certain characteristics were independently associated with significant BP changes from the mean BP change. Several previous multivariate mixed model analyses, including patient-level data published to date, found no consistent characteristic associated with BP response aside from baseline BP.^18^, ^19^, ^20^ These analyses, however, differ in several ways from the present study including having a smaller sample size, a shorter follow-up period, and varied statistical approaches.

### Biphasic BP reduction hypotheses

Modeled estimated systolic and diastolic BP reductions were biphasic with an initial steeper reduction post-RDN followed by a persistent yet shallower BP decline. The initial BP reduction presumably reflects acute modulation of sympathetic nervous system activity between the kidneys and the central nervous system.^21^, ^22^, ^23^ There was a consistent BP lowering observed through 3 years of follow-up. Moreover, persistent BP reductions through long-term follow-up out to 10 years have been previously reported.^11^^,^^18^^,^^19^^,^^24^, ^25^, ^26^, ^27^, ^28^, ^29^, ^30^, ^31^, ^32^, ^33^, ^34^, ^35^, ^36^, ^37^, ^38^ These data are supported by preclinical work in several large animal models demonstrating that renal sympathetic nerves do not functionally recover following RF or surgical RDN.^39^^,^^40^ Singh and colleagues showed some evidence of partial functional recovery although BP reduction was also sustained through 30 months in an ovine CKD model.^41^ A key factor contributing to the lack of functional recovery is the absence of myelination of renal nerves. Consequently, nerve atrophy and axonal loss persist distal to the site of ablation following RDN. This sustained damage impairs the ability of the nerves to regain their normal function, highlighting the long-term effects of the procedure on nerve integrity.

Several possible mechanistic reasons might explain the continued BP reductions following the procedure. One mechanism that can be ruled out is an increase in medication classes over time, as this did not change from baseline. A common misconception is that RDN is expected to reduce the number of AH medications. However, no trial has systematically assessed whether medication can be stopped or down-titrated following RDN. Consistent with U.S. Food and Drug Administration approval of RDN for the treatment of uncontrolled hypertension and recent guidelines, RDN should be considered the third pillar of treatment options in addition to lifestyle changes and AH medications.^6^^,^^42^ It is possible that there is an interaction between RDN and specific AH drug classes, which in combination may reduce BP synergistically beyond what would be expected for each therapy individually.^43^ Although, a recent study from the GSR did not find an interaction between the number of antihypertension classes prescribed nor the prescription of aldosterone antagonists,^11^ herein, aldosterone antagonist use was associated with greater falls in BP. RDN, in addition to mineralocorticoid receptor antagonists, might provide further neural hormonal blockade in patients with difficult to control BP.^44^ One other possibility is modulating sympathetic tone from the kidneys interacts with the activity of the renin-angiotensin-aldosterone system, which may abate overtime following RDN, thus further reducing BP particularly when AH medication is added.^43^^,^^45^ Another possibility is that decreased central sympathetic activity may reduce peripheral vascular resistance, thereby lowering BP further over time.^46^ Alternatively, decrease or cessation of renal sympathetic tone following RDN may interrupt and reset the baroreflex. RDN with treatment of the branch arteries has been shown to modulate other aspects related to altering the afferent sympathetic nervous system activity, such as reducing heart rate or aldosterone concentration.^47^^,^^48^ Furthermore, another plausible mechanism for continued BP reductions over time is the reversal of vascular hypertrophy following RDN.^49^ A similar phenomenon has been observed by the blockade of angiotensin II receptors.^50^ Each of these hypotheses are not mutually exclusive and may individually or in combination explain the continuous BP reductions after RDN through long-term follow-up. Further long-term investigations will help elucidate the potential mechanisms underlying continuous BP reductions following RDN.

### Patient and procedural characteristics associated with BP changes from the model

Several different baseline patient characteristics were associated with office and/or 24-h systolic and diastolic BP reductions. Higher baseline BP was the most consistent characteristic associated with large BP reductions for the corresponding dependent BP measure at follow-up. For example, higher baseline office SBP was associated with larger office SBP reductions. Notably, the remainder of these individual characteristic-associated BP changes were modest (<2 mm Hg). While no single patient characteristic was consistent for all BP measures aside from baseline BP, several characteristics were repeatedly associated with greater BP reductions in the models and are comorbidities consistent with higher sympathetic activity. For example, type 2 diabetes has been linked to higher sympathetic activity,^51^ particularly during early stages of the disease.^52^ Atrial fibrillation is associated with increased sympathetic nervous system activity that can indeed be modulated by RDN via ablation afferent renal nerves.^53^^,^^54^ Thus, history of atrial fibrillation may serve as a surrogate marker for increased sympathetic tone making those patients potential candidates for RDN. Age and history of heart failure were also significantly correlated with greater reductions in several BP measures. The association between age and cardiovascular events with increased sympathetic nervous system activity is well established.^4^^,^^55^, ^56^, ^57^ Heart failure, irrespective of left ventricular ejection fraction, has been characterized by increased sympathetic nervous system activity.^58^ Importantly, multiple different characteristics associated with a significant BP change may have an additive effect. Interestingly, higher baseline eGFR in some models and higher serum creatinine in other models were associated with greater BP reductions. These seemingly contradictory outcomes may be indicative that RDN represents a suitable therapy for patients with hypertension independent of renal function. In other studies, the presence of CKD was not associated with worse response to RDN.^59^

### Study Limitations

There are several limitations to this analysis. The pooled, patient-level data come from a variety of trials with different designs, inclusion/exclusion criteria. SYMPLICITY HTN-3, SPYRAL HTN-OFF MED, and SPYRAL HTN-ON MED were randomized, sham-controlled trials with strict enrollment criteria, although varying prescribed AH medications, whereas GSR is open-label, all-comers, real-world registry without stringent inclusion criteria. The more covariates included in the model necessarily increase the statistical chance that some characteristic may emerge as being significantly associated with BP change, but without an underlying physiological or mechanistic basis, which is an inherent limitation of such a model. Moreover, some procedural characteristics were not collected across all trials and thus could not be pooled. Any retrospectively identified parameter should be validated in prospectively planed studies. It must be emphasized that characteristics identified in the multivariate mixed model and discussed here are not necessarily “predictors” of response to RDN. The model does not indicate the strength of the correlated BP changes at follow-up, only whether the changes are statistically significant. Nor are reductions sham adjusted. Thus, results should be interpreted as hypothesis-generating, with the aim of exploring particular patient characteristics of interest in future, prospective trials with adequate assessment of predictive accuracy.

## Conclusions

This multivariate mixed model analysis including over 4,155 patients and more than 12,000 years of patient-level data identified multiple patient characteristics associated with increased sympathetic activity. Higher baseline BP was the most significant and consistent contributor to BP across all measures. Further studies are required to validate whether any particular characteristic is prospectively associated with improved BP outcomes. The model indicated durable biphasic BP reductions following RDN, with a steeper immediate reduction followed by a steady decline through 3 years.

## Funding Source and Author Disclosures

This study was funded by Medtronic. Dr Mahfoud is supported by 10.13039/501100010578Deutsche Gesellschaft für Kardiologie (DGK), Deutsche Forschungsgemeinschaft (SFB TRR219, Project-ID 322900939), and Deutsche Herzstiftung. Saarland University has received scientific support from Ablative Solutions, Medtronic, and ReCor Medical. Until May 2024, Dr Mahfoud has received speaker honoraria/consulting fees from Ablative Solutions, Amgen, AstraZeneca, Bayer, Boehringer Ingelheim, Inari, Medtronic, Merck, ReCor Medical, Servier, and Terumo. Dr Townsend is a consultant for Medtronic, Axio, Regeneron, Bard, OBIO, and AstraZeneca; and has received royalties from UpToDate. Dr Kandzari has received institutional research/grant support from Biotronik, Boston Scientific, Orbus Neich, Teleflex, Medtronic, and Ablative Solutions; he also has received personal consulting honoraria from Medtronic and HyperQure. Dr Mancia has received speaker honoraria from Berlin Chemie, Exicon Conshlting, Menarini Int, Merck Healthcare KGaA, Medtronic Inc USA, Recordati, Sanofi, Servier, and Sun Laboratories. Dr Lauder has received speaker honoraria/consulting fees from AstraZeneca, Medtronic, Pfizer, and ReCor Medical. Dr Bhatt is on the advisory board for Angiowave, Bayer, Boehringer Ingelheim, Cardax, CellProthera, Cereno Scientific, Elsevier Practice Update Cardiology, High Enroll, Janssen, Level Ex, McKinsey, Medscape Cardiology, Merck, MyoKardia, NirvaMed, Novo Nordisk, PhaseBio, PLx Pharma, and Stasys; is on the board of directors for American Heart Association New York City, Angiowave (stock options), Bristol Myers Squibb (stock), DRS.LINQ (stock options), and High Enroll (stock); is a consultant for Broadview Ventures, Hims; is on the data monitoring committees for Acesion Pharma, Assistance Publique-Hôpitaux de Paris, Baim Institute for Clinical Research (formerly Harvard Clinical Research Institute, for the PORTICO trial, funded by 10.13039/100006279St. Jude Medical, now Abbott), Boston Scientific (Chair, PEITHO trial), Cleveland Clinic, Contego Medical (Chair, PERFORMANCE 2), Duke Clinical Research Institute, Mayo Clinic, Mount Sinai School of Medicine (for the ENVISAGE trial, funded by Daiichi Sankyo; for the ABILITY-DM trial, funded by Concept Medical), Novartis, Population Health Research Institute; Rutgers University (for the NIH-funded MINT Trial); has received honoraria from American College of Cardiology (Senior Associate Editor, Clinical Trials and News, ACC.org; Chair, ACC Accreditation Oversight Committee), Arnold and Porter law firm (work related to Sanofi/Bristol-Myers Squibb clopidogrel litigation), Baim Institute for Clinical Research (formerly Harvard Clinical Research Institute; RE-DUAL PCI clinical trial steering committee funded by Boehringer Ingelheim; AEGIS-II executive committee funded by CSL Behring), Belvoir Publications (Editor in Chief, Harvard Heart Letter), Canadian Medical and Surgical Knowledge Translation Research Group (clinical trial steering committees), CSL Behring (AHA lecture), Cowen and Company, Duke Clinical Research Institute (clinical trial steering committees, including for the PRONOUNCE trial, funded by Ferring Pharmaceuticals), HMP Global (Editor in Chief, Journal of Invasive Cardiology), *Journal of the American College of Cardiology* (Guest Editor; Associate Editor), K2P (Co-Chair, interdisciplinary curriculum), Level Ex, Medtelligence/ReachMD (CME steering committees), MJH Life Sciences, Oakstone CME (Course Director, Comprehensive Review of Interventional Cardiology), Piper Sandler, Population Health Research Institute (for the COMPASS operations committee, publications committee, steering committee, and USA national co-leader, funded by Bayer), WebMD (CME steering committees), Wiley (steering committee); other services from Clinical Cardiology (Deputy Editor); holds a patent on Sotagliflozin (named on a patent for sotagliflozin assigned to Brigham and Women's Hospital who assigned to Lexicon; neither I nor Brigham and Women's Hospital receive any income from this patent); has received research funding from Abbott, Acesion Pharma, Afimmune, Aker Biomarine, Alnylam, Amarin, Amgen, AstraZeneca, Bayer, Beren, Boehringer Ingelheim, Boston Scientific, Bristol Myers Squibb, Cardax, CellProthera, Cereno Scientific, Chiesi, CinCor, Cleerly, CSL Behring, Eisai, Ethicon, Faraday Pharmaceuticals, Ferring Pharmaceuticals, Forest Laboratories, Fractyl, Garmin, HLS Therapeutics, Idorsia, Ironwood, Ischemix, Janssen, Javelin, Lexicon, Lilly, Medtronic, Merck, Moderna, MyoKardia, NirvaMed, Novartis, Novo Nordisk, Otsuka, Owkin, Pfizer, PhaseBio, PLx Pharma, Recardio, Regeneron, Reid Hoffman Foundation, Roche, Sanofi, Stasys, Synaptic, The Medicines Company, Youngene, 89Bio; has received royalties from Elsevier (Editor, Braunwald's Heart Disease); is a site co-investigator for Abbott, Biotronik, Boston Scientific, CSI, Endotronix, St. Jude Medical (now Abbott), Philips, SpectraWAVE, Svelte, Vascular Solutions; is a trustee of the American College of Cardiology; and has done unfunded research for FlowCo. Dr Kario has received personal fees from Medtronic; has received grants from A&D Company, JIMRO, Omron Healthcare, CureApp, Terumo, and Fukuda Denshi; has received honoraria from Otsuka Pharmaceuticals and Omron Healthcare, and has participated on the advisory board of Fukuda Denshi outside the submitted work. Dr Schmieder has received speaker and consulting honoraria from Medtronic, Recor, and Ablative solutions. Research grants have been given to his institution from Medtronic, Recor, and Ablative solutions. Dr Fahy was an employee of Medtronic. Dr Böhm is supported by the Deutsche Forschungsgemeinshaft (SFB TTR219); and has received personal fees from Abbott, Amgen, AstraZeneca, Bayer, Boehringer Ingelheim, Cytokinetics, Medtronic, Novartis, ReCor Medical, Servier, and Vifor. All other authors have reported that they have no relationships relevant to the contents of this paper to disclose.

## Supplementary data

Supplemental Tables 1, 2, 3, 4, 5, 6, 7, 8, ,9, 10, and 11, and Supplemental Figures 1, 2, and 3
