# Supplementary material for: Long-Term, Patient-Level Analysis of Radiofrequency Renal Denervation in the SYMPLICITY Clinical Trial Program
Source: JACC Adv. 2025 Feb 21;4(3):101606. doi: 10.1016/j.jacadv.2025.101606 (PMC11904547; doi:10.1016/j.jacadv.2025.101606)
Supplement: Supplemental Tables 1, 2, 3, 4, 5, 6, 7, 8, ,9, 10, and 11, and Supplemental Figures 1, 2, and 3 [file mmc1.docx]

**Supplemental Table 1. Antihypertensive medication classes prescribed at baseline in patients from the Pooled SYMPLICITY RDN program**

| **Baseline antihypertensive medication class** | **% (n/N)** |
| --- | --- |
| Aldosterone antagonist | 24.5% (896/3651) |
| Alpha 1 blocker* | 7.2% (59/822) |
| Alpha 2 antagonist* | 25.5% (210/822) |
| ACE inhibitor | 33.6% (1227/3651) |
| Angiotensin receptor blocker | 59.1% (2156/3651) |
| Beta blocker | 71.2% (2601/3651) |
| Calcium channel blocker | 72.4% (2644/3651) |
| Diuretic | 74.1% (2706/3651) |
| Direct renin inhibitor | 4.8% (176/3651) |
| Vasodilator | 14.6% (533/3651) |

*Data collection on indicated medication class was not collected in GSR DEFINE.

Supplemental Table 2. Procedural characteristics from the SYMPLICITY RDN program

| Procedural characteristic | Mean ± SD |
| --- | --- |
| Procedure duration (min) | 84.4 ± 43.4 |
| Catheter duration (min) | 47.9 ± 22.1 |
| Contrast volume (mL) | 143.2 ± 84.1 |
| Number of ablation attempts | 19.7 ± 16.8 |

Data include Flex and Spyral patients.Supplemental Table 3. Characteristics associated with BP changes across BP measures in the Flex & Spyral models

| **Patient and procedural characteristics** | **Office systolic BP change from baseline (mmHg)** | **24-hr systolic BP change from baseline (mmHg)** | **Office diastolic BP change from baseline (mmHg)** | **24-hr diastolic BP change from baseline (mmHg)** |
| --- | --- | --- | --- | --- |
| Baseline office systolic BP (+10 mmHg) | -4.52 | 0.82 | -0.31 | 0.18 |
| Baseline 24-hr ambulatory systolic BP (+10 mmHg) | NS | -3.59 | NS | -0.51 |
| Baseline office diastolic BP (+10 mmHg) | NS | NS | -3.71 | NS |
| Baseline 24-hr ambulatory diastolic BP (+10 mmHg) | NS | NS | NS | -2.41 |
| Number of AH medications at baseline | 0.51 | 1.29 | 0.59 | 0.96 |
| Number of AH medications through follow-up | NS | -0.89 | -0.41 | -0.81 |
| History of heart failure | -1.64 | NS | -1.15 | NS |
| History of atrial fibrillation | -1.54 | NS | NS | NS |
| Combined hypertension | -1.16 | -1.79 | NS | 0.86 |
| Baseline serum creatinine (+1mg/dL) | -0.85 | NS | -0.72 | NS |
| Vasodilator prescription | 3.09 | NS | NS | NS |
| Increased age (+1 year) | NS | -0.05 | -0.16 | -0.07 |
| Aldosterone antagonist prescription | NS | -1.32 | NS | -0.73 |
| Baseline eGFR (+1 mL/min/1.73m^2^) | NS | -0.02 | NS | NS |
| Types 2 diabetes | NS | NS | -1.55 | -0.61 |
| Contrast volume (+10 mL) | NS | NS | 0.04 | NS |

Significant changes shown in mmHg. NS = non-significant. AH = antihypertensive. Characteristics that were non-significant for all BP measures are excluded.

Supplemental Table 4. Modeling results for fixed effects on office diastolic blood pressure change through 36 months

| **Fixed effect** | **DBP change estimate (mmHg)** | **95% CI (mmHg)** | **Pr > \|t\|** |
| --- | --- | --- | --- |
| Baseline office diastolic BP (+10 mmHg) | -3.71 | -3.93, -3.49 | <0.0001 |
| Baseline office systolic BP at baseline (+10 mmHg) | -0.31 | -0.44,-0.17 | <0.0001 |
| No. of mediations at baseline | 0.59 | 0.39,0.79 | <0.0001 |
| No. of medications thru follow-up | -0.41 | -0.59,-0.23 | <0.0001 |
| Increased age (per year) | -0.16 | -0.19,-0.14 | <0.0001 |
| Type 2 diabetes | -1.55 | -2.10,-1.00 | <0.0001 |
| History of heart failure | -1.15 | -1.96,-0.34 | 0.0055 |
| Baseline serum creatinine | -0.72 | -1.04,-0.39 | <0.0001 |
| Contrast volume (+10 mL) | 0.04 | 0.006,0.07 | 0.018 |
| Follow-up |  |  |  |
| Baseline | reference | - | - |
| 3-months | -4.67 | -5.16,-4.17 | <0.0001 |
| 6-months | -5.46 | -5.96,-4.96 | <0.0001 |
| 12-months | -6.12 | -6.63,-5.62 | <0.0001 |
| 24-months | -7.21 | -7.76,-6.67 | <0.0001 |
| 36-months | -7.81 | -8.39,-7.24 | <0.0001 |

Non-significant covariates excluded.

Supplemental Table 5. Modeling results for fixed effects on 24-h ambulatory diastolic blood pressure change through 36 months

| **Fixed effect** | **DBP change estimate (mmHg)** | **95% CI (mmHg)** | **Pr > \|t\|** |
| --- | --- | --- | --- |
| Baseline 24-h ambulatory diastolic BP (+10 mmHg) | -2.41 | -2.68,-2.15 | <0.0001 |
| Baseline 24-h ambulatory systolic BP (+10 mmHg) | -0.51 | -0.71,-0.32 | <0.0001 |
| Baseline office systolic BP (+10 mmHg) | 0.18 | 0.05,0.30 | 0.0056 |
| No. of mediations at baseline | 0.96 | 0.77,1.15 | <0.0001 |
| No. of medications thru follow-up | -0.81 | -0.98,-0.64 | <0.0001 |
| Increased age (per year) | -0.07 | -0.10,-0.05 | <0.0001 |
| Type 2 diabetes | -0.61 | -1.11,-0.12 | 0.014 |
| Combined hypertension | 0.86 | 0.23,1.48 | 0.0072 |
| Prescribed aldosterone antagonist | -0.73 | -1.33,-0.14 | 0.016 |
| Follow-up |  |  |  |
| Baseline | reference | - | - |
| 3-months | -4.03 | -4.49,-3.57 | <0.0001 |
| 6-months | -4.86 | -5.28,-4.43 | <0.0001 |
| 12-months | -5.46 | -5.90,-5.01 | <0.0001 |
| 24-months | -6.40 | -6.90,-5.90 | <0.0001 |
| 36-months | -6.99 | -7.54,-6.43 | <0.0001 |

Non-significant covariates excluded.

Supplemental Table 6. Modeling results for fixed effects on office systolic blood pressure change through 36 months in Spyral patients

| **Fixed effect** | **DBP change estimate (mmHg)** | **95% CI (mmHg)** | **Pr > \|t\|** |
| --- | --- | --- | --- |
| Baseline office systolic BP (+10 mmHg) | -3.48 | -3.78,-3.18 | <0.0001 |
| No. of mediations at baseline | 1.82 | 1.33,2.30 | <0.0001 |
| No. of medications thru follow-up | -1.49 | -1.97,-1.00 | <0.0001 |
| History of atrial fibrillation | -2.40 | -4.74,-0.05 | 0.045 |
| Baseline serum creatinine | -0.68 | -1.29,-0.06 | 0.031 |
| Follow-up |  |  |  |
| Baseline | reference | - | - |
| 3-months | -10.56 | -11.82,-9.30 | <0.0001 |
| 6-months | -13.73 | -15.01,-12.45 | <0.0001 |
| 12-months | -14.94 | -16.26,-13.63 | <0.0001 |
| 24-months | -15.76 | -17.22,-14.31 | <0.0001 |
| 36-months | -17.34 | -18.94,-15.75 | <0.0001 |

Non-significant covariates excluded.

Supplemental Table 7. Modeling results for fixed effects on 24-h ambulatory systolic blood pressure through 36 months for Spyral patients

| **Fixed effect** | **SBP estimate (mmHg)** | **95% CI (mmHg)** | **Pr > \|t\|** |
| --- | --- | --- | --- |
| Baseline 24-h ambulatory systolic BP (+10 mmHg) | -3.42 | -3.90,-2.94 | <0.0001 |
| Baseline office systolic BP (+10 mmHg) | 0.85 | 0.43,1.28 | <0.0001 |
| No. of mediations at baseline | 1.75 | 1.26,2.23 | <0.0001 |
| No. of medications thru follow-up | -1.71 | -2.19,-1.24 | <0.0001 |
| BMI | 0.16 | 0.06,0.26 | 0.0018 |
| Combined hypertension | -3.18 | -4.97,-1.40 | 0.0005 |
| Baseline potassium (mmol/L)* | -1.33 | -2.59,-0.07 | 0.038 |
| Number of ablation attempts (+4)† | -0.14 | -0.26,-0.02 | 0.023 |
| Follow-up |  |  |  |
| Baseline | reference | - | - |
| 3-months | -6.78 | -7.96,-5.59 | <0.0001 |
| 6-months | -9.18 | -10.38,-7.97 | <0.0001 |
| 12-months | -10.27 | -11.51,-9.03 | <0.0001 |
| 24-months | -11.45 | -12.83,-10.06 | <0.0001 |
| 36-months | -13.48 | -15.00,-11.95 | <0.0001 |

Non-significant covariates excluded. *SPYRAL FIM not included in analysis. † Each placement of the SPYRAL catheter equals up to 4 ablations.

Supplemental Table 8. Modeling results for fixed effects on office diastolic blood pressure change through 36 months for Spyral patients

| **Fixed effect** | **DBP change estimate (mmHg)** | **95% CI (mmHg)** | **Pr > \|t\|** |
| --- | --- | --- | --- |
| Baseline office diastolic BP at baseline (+10 mmHg) | -3.53 | -3.87,-3.18 | <0.0001 |
| No. of mediations at baseline | 0.97 | 0.64,1.29 | <0.0001 |
| No. of medications thru follow-up | -1.14 | -1.45,-0.82 | <0.0001 |
| Increased age (per year) | -0.19 | -0.23,-0.14 | <0.0001 |
| Baseline eGFR (mL/min/1.73m^2^) | -0.04 | -0.06,-0.01 | 0.0069 |
| Baseline serum creatinine (mg/dL) | -1.18 | -1.70,-0.67 | <0.0001 |
| Baseline potassium (mmol/L)* | -1.23 | -2.17,-0.30 | 0.0095 |
| Contrast volume (+10 mL) | 0.06 | 0.01,0.11 | 0.011 |
| Follow-up |  |  |  |
| Baseline | reference | - | - |
| 3-months | -5.35 | -6.20,-4.50 | <0.0001 |
| 6-months | -6.65 | -7.51,-5.80 | <0.0001 |
| 12-months | -7.26 | -8.12,-6.39 | <0.0001 |
| 24-months | -8.16 | -9.10,-7.22 | <0.0001 |
| 36-months | -8.56 | -9.56,-7.56 | <0.0001 |

Non-significant covariates excluded. *SPYRAL FIM not included in analysis.

Supplemental Table 9. Modeling results for fixed effects on 24-hr ambulatory diastolic blood pressure change through 36 months for Spyral patients

| **Fixed effect** | **DBP change estimate (mmHg)** | **95% CI (mmHg)** | **Pr > \|t\|** |
| --- | --- | --- | --- |
| Baseline 24-h ambulatory diastolic BP (+10 mmHg) | -2.26 | -2.58,-1.94 | <0.0001 |
| No. of mediations at baseline | 1.39 | 1.10,1.69 | <0.0001 |
| No. of medications thru follow-up | -1.55 | -1.84,-1.26 | <0.0001 |
| Baseline potassium (mmol/L)* | -1.28 | -2.08,-0.48 | 0.0018 |
| Contrast volume (+10 mL) | 0.05 | 0.01,0.08 | 0.025 |
| Follow-up |  |  |  |
| Baseline | reference | - | - |
| 3-months | -4.34 | -5.07,-3.62 | <0.0001 |
| 6-months | -5.82 | -6.56,-5.08 | <0.0001 |
| 12-months | -6.52 | -7.28,-5.76 | <0.0001 |
| 24-months | -7.35 | -8.20,-6.50 | <0.0001 |
| 36-months | -8.45 | -9.38,-7.52 | <0.0001 |

Non-significant covariates not shown. *SPYRAL FIM not included in analysis.

**Supplemental Table 10. Clinical outcomes through 3 years**

|  | All patients (2,262) | Spyral patients only (743) |
| --- | --- | --- |
| Renal artery stenosis | 0.3% (6) | 0% (0) |
| All cause death | 5.1% (134) | 5.1% (38) |
| Cardiac death | 2.8% (73) | 2.3% (17) |
| Myocardial infarction | 2.7% (70) | 1.5% (11) |
| Stroke | 4.5% (118) | 4.4% (33) |
| Hospitalization for hypertensive crisis | 3.5% (93) | 2.3% (17) |

Supplemental Table 11. Clinical outcomes through 1 year

|  | All patients (3,604) | Spyral patients (1192) |
| --- | --- | --- |
| Renal artery stenosis | 0.1% (3) | 0% (0) |
| All cause death | 1.2% (45) | 0.9% (11) |
| Cardiac death | 0.7% (26) | 0.4% (5) |
| Myocardial infarction | 0.9% (34) | 0.1% (1) |
| Stroke | 1.4% (49) | 1.3% (16) |
| Hospitalization for hypertensive crisis | 1.4% (52) | 0.9% (11) |


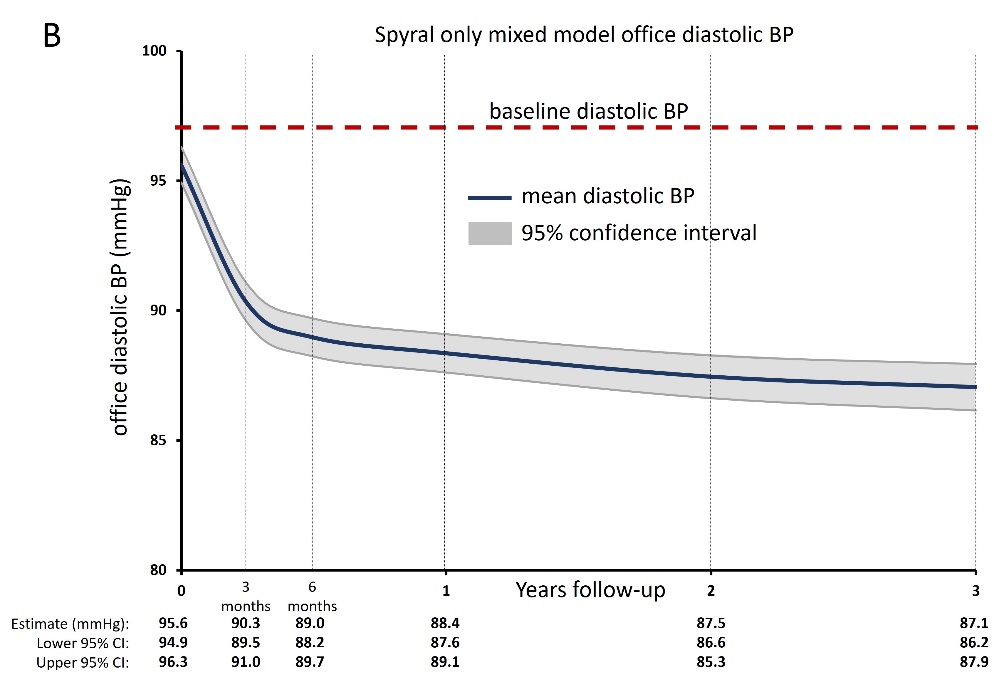

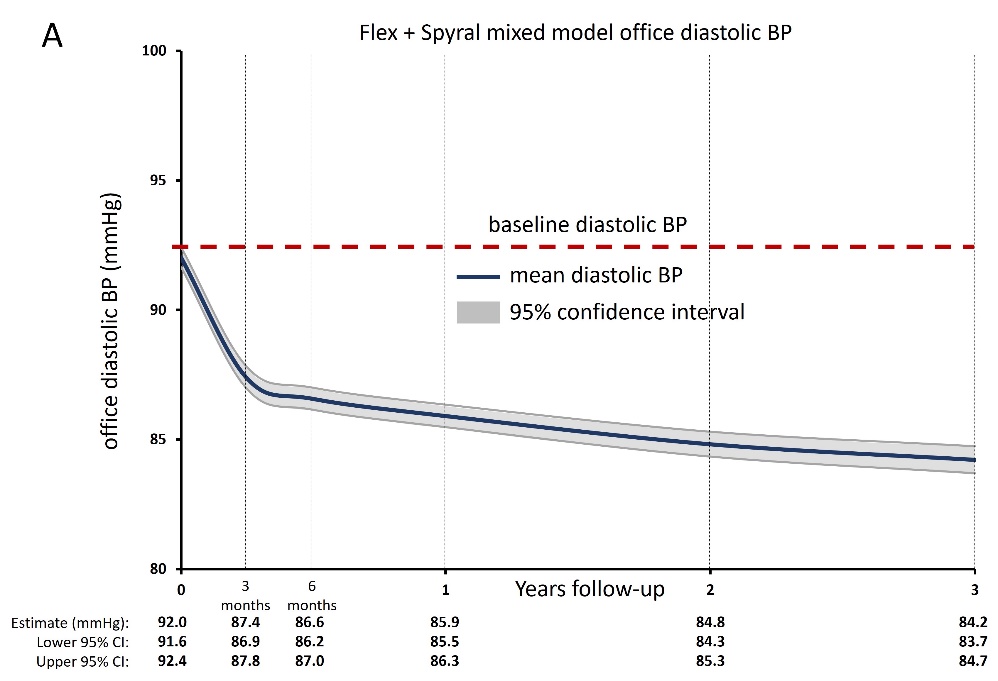
Supplemental Figure 1. Mixed Model Office Diastolic BP Estimates after RF RDN using the Symplicity RF RDN System through 36 Months

Least square means estimates of office diastolic BP from baseline through 36 months after RF RDN from patients treated with either the (A) Flex and Spyral catheter or (B) the Spyral catheter only.


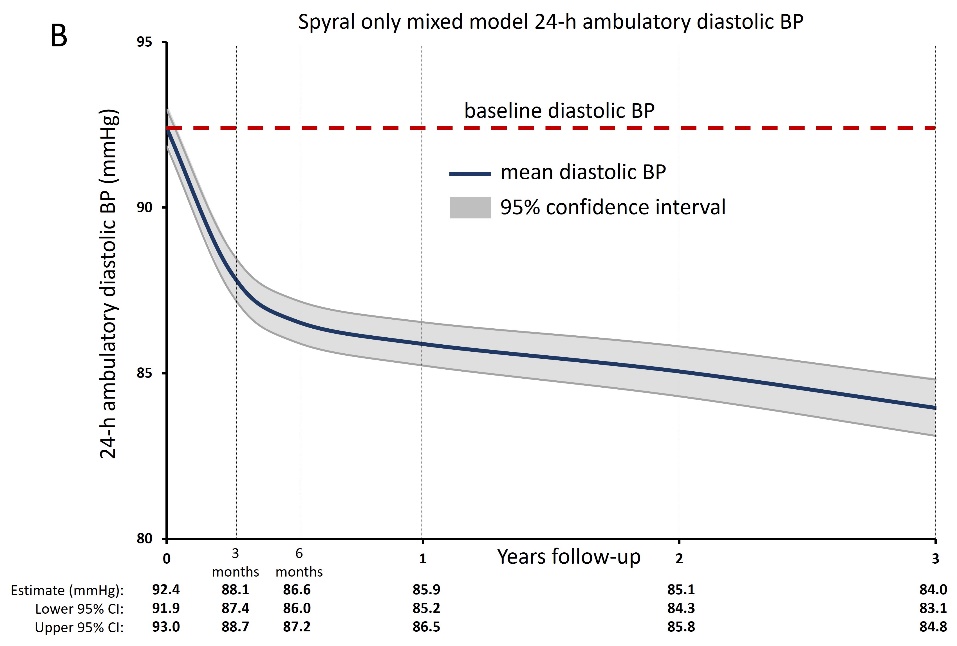

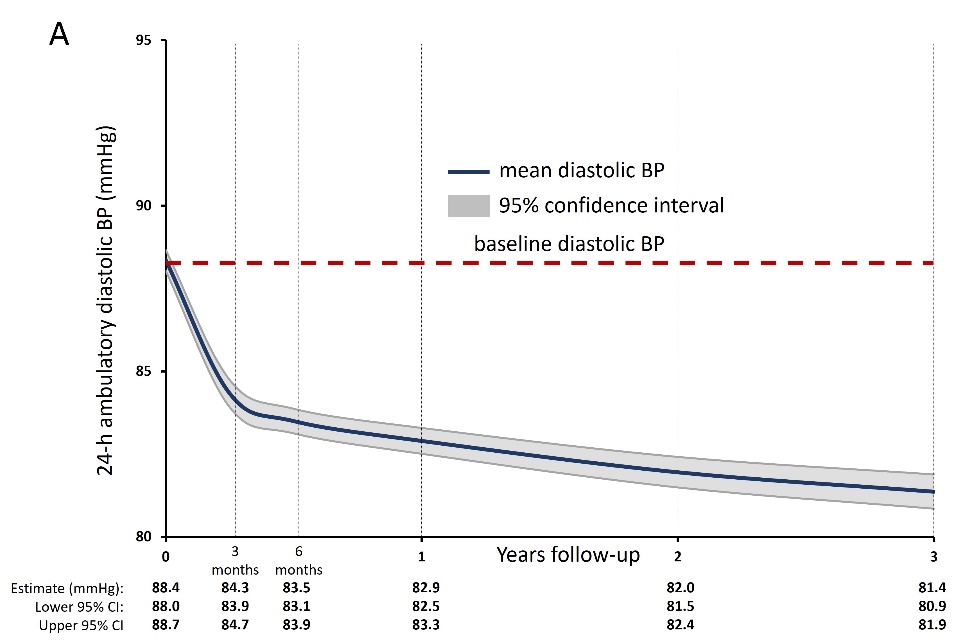
Supplemental Figure 2. Mixed Model 24-h Ambulatory Diastolic BP Estimates after RF RDN using the Spyral Catheter through 36 Months

Least square means estimates of 24-h ambulatory diastolic BP from baseline through 36 months after RF RDN from patients treated with either the (A) Flex and Spyral catheter or (B) the Spyral catheter only.

**Supplemental Figure 3. The change in mean office and 24-h ambulatory pulse pressure among all patients from baseline through 36 months**


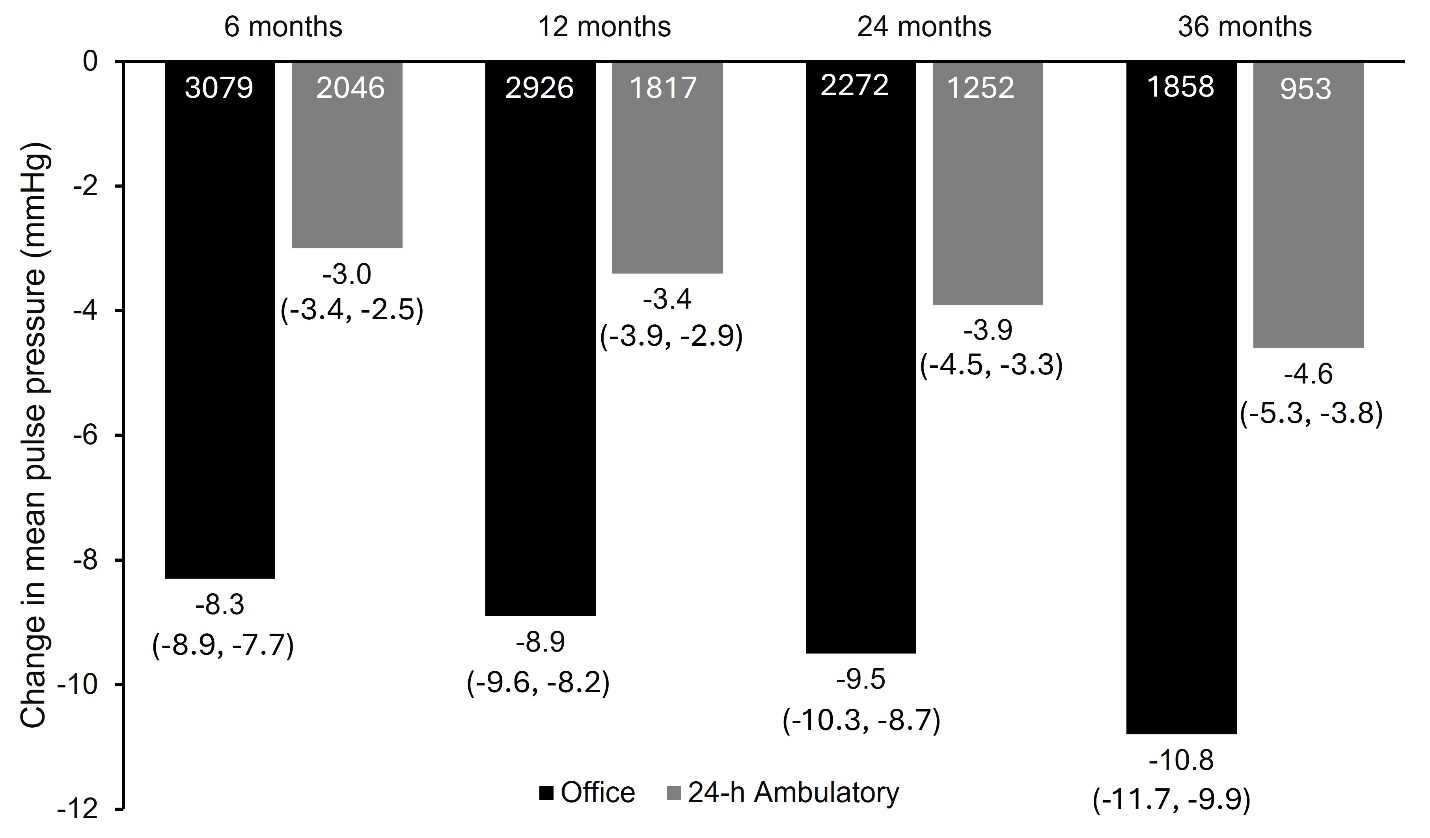


Mean pulse pressure reduction (95% confidence interval) are reported.
